# 

**Published:** 1888-02

**Authors:** 


					﻿Vol VIII.
FEBRUARY, 1888.
No. 2.
THE
HOMEOPATHIC PHYSICIAN,
A Monthly Journal of Homoeopathic Materia Medica
and Clinical Medicine.
“ He is a freeman whom truth makes free, and all are slaves beside.”
“ Seek the Truth: come whence it may, cost what it will.”
EDITED AND PFBLI8HED BY
Edmund j.	Nl*
AND.
WALTER M. JAMES, M. D.
PHILADELPHIA :
No. 1123 SPRUCE 8TREET.
LONDO 3TS
ALFRED HEATH & CO., 8ox.e Agents fob Great Britain,
114 Ebury Street, Eaton Square.
Yearly Subscription, $2.SO, invariably in advance. Single numbers, 25 cts.
Entered at the Philadelphia PoevOffiae aa Second-Clan Mail Matter.
				

